# Retrospective cohort analysis of heart rate variability in patients with high altitude pulmonary hypertension in Tibet

**DOI:** 10.1002/clc.23312

**Published:** 2019-12-19

**Authors:** Zhang Qian, Aili Fan, Dawa, Dawaciren, Binbin Pan

**Affiliations:** ^1^ Heart Center Peking University People's Hospital Beijing China; ^2^ Department of High Altitude Sickness and Cardiovascular Disease The People's Hospital of the Tibet Autonomous Region Heart Center Tibet Lisa China

**Keywords:** autonomic nervous system, heart rate variability, high altitude, high altitude pulmonary hypertension

## Abstract

**Background:**

Studies from both humans and animals experiments have offered abundant evidence supporting that mountain sickness is associated with changes in autonomic nervous function (ANF), which can be measured by heart rate variability (HRV).

**Hypothesis:**

We aimed to assess changes of ANF in chronic mountain disease by measuring HRV in patients with high altitude pulmonary hypertension (HAPH).

**Methods:**

From November 2018 to March 2019, 120 patients in the cardiac care unit of the People's Hospital of Tibet Autonomous Region were selected as the observation group, and 50 patients without organic heart disease served as the control group. Pulmonary artery systolic pressure was evaluated by echocardiography in patients with HAPH, divided into three groups: mild (30‐49 mm Hg), moderate (50‐69 mm Hg) and severity (≥70 mm Hg) groups. A 24‐hour dynamic electrocardiogram (DCG) was obtained for each patient. HRV (SDNN, SDANN, RMSSD, PNN50, and HRVTI for time domain; TP, VLF, LF, HF, and LF/HF for frequency domain) indexes were measured and compared.

**Results:**

Compared with the control group, time domain parameters including SDNN, SDANN, RMSSD, PNN50, and HRVTI were reduced, as well as frequency domain indexes such as TP, VLF, LF, and HF. LF/HF was highest in mild HAPH group and lowest in the moderate HAPH group, and the difference between the two groups was statistically significant.

**Conclusions:**

The HRV of patients with chronic HAPH in high altitude areas in Tibet is significantly reduced relative to healthy controls, and significantly negatively correlated with the severity of pulmonary artery hypertension.

## 1 INTRODUCTION

Chronic mountain sickness is a disease of high‐altitude residents whose most common causes of death are chronic pulmonary hypertension and right heart failure. High‐altitude‐pulmonary hypertension (HAPH) is a specific disease affecting individuals at altitudes above 2500 m. The VI World Congress on Mountain Medicine & High Altitude Physiology unified it as high altitude pulmonary hypertension (HAPH).^1^ Studies by Wu Tian Yi et al showed that the prevalence of HAPH is higher in children than adults in Qinghai, China, and the difference is more obvious with the increase of altitude.^2^ There are currently no relevant clinical studies focusing on autonomic changes in HAPH. Research is limited to changes in ANF in acute mountain sickness. In the development of HAPH, due to the low‐oxygen hypobaric environment and pulmonary vascular remodeling, the right heart load is aggravated, affecting the sympathetic and vagus nerves that innervate the heart, and disrupting the balance of cardiac autonomic nervous system regulation. This leads to arrhythmia, cardiac disease, and sudden death. Therefore, it is imperative to explore ANF for chronic mountain disease, especially chronic HAPH.

## 2 MATERIALS AND METHODS

### 2.1 Ethics statement

The current survey was officially implemented after approval from the Medical Ethics Committee of the People's Hospital of Tibet Autonomous Region, China (approval number, ME‐TBHP‐36). All subjects or their families fully understood the purpose and methods of the study, and provided signed informed consent. There were no health interventions involving the subjects. All individual data were anonymous prior to analysis.

### 2.2 Study subjects and protocol

From November 2018 to May 2019, 120 patients with HAPH were admitted to the Cardiac Care Unit of the People's Hospital of Tibet Autonomous Region, including 64 males, aged 22‐84 (63.78 ± 1.56) years, and 56 females, aged 28‐84 (63.34 ± 1.33) years. Those taking calcium antagonists and β blockers affecting HRV were excluded. All participants provided written informed consent. All subjects were questioned about ethnicity, occupation, living altitude and detailed medical history after hospitalization, and underwent physical examination. Hemoglobin measurement, electrocardiography, 24‐hour dynamic electrocardiography (DCG), and echocardiography were performed within 48 hours of admission. Also, finger oxygen saturation (SaO_2_) levels were measured by hand‐held oxygen meter (TuffSat; GE Healthcare, London, England) in the second left finger. The control group consisted of 50 patients with no definite cardiopulmonary disease and no signs of pulmonary hypertension on echocardiography. The subjects were all Tibetans living at altitudes >3000 m. The baseline data of each group of subjects are shown in Table 1.

**Table 1:** Clinical characteristics of the patients

Index	Control N = 50	Mild HAPH N = 40	Moderate HAPH N = 40	Severe HAPH N = 40	Total N = 170
Male:female	21:28	23:17	19:21	22:18	86:84
Age (years)	59.44 ± 0.73	59.95 ± 2. 10	61.05 ± 1.44	62.73 ± 1.56	62.36 ± 10.08
Current residence					
4500 (Ngari)	10	2	7	1	20
3500 (Changdu)	5	2	6	6	21
3658 (Lhasa)	11	12	10	6	39
3000 (Lin Zhi)	4	5	6	5	20
4507 (Nagqu)	8	1	4	14	27
3836 (Shigatse)	5	10	2	5	22
3700 (Shannan)	7	6	5	3	21
Living altitude (meters)	3917.48 ± 68.56	3674.08 ± 55.01	3782.00 ± 80.27	3895.65 ± 84.05	3823.19 ± 36.81
Education					
Primary	1	9	7	3	20
Junior High	37	25	18	25	105
Senior High	11	5	14	9	39
University	1	2	1	3	6
Smoking status					
Nonsmoker	16	8	8	9	41
Current smoker	31	30	28	29	118
Former smoker	3	2	4	2	11
Duration of diagnosis (years)	non	4.30 ± 0.63	3.65 ± 1.78	8.78 ± 2.61**	5.00 ± 0.65
Average heart rate (prm)	67.70 ± 8.13	65.4 ± 8.17*	61.33 ± 4.49*	60.05 ± 5.92*	63.86 ± 7.86
PAP， mm Hg	24.96 ± 0.28	43.05 ± 0.77**	59.10 ± 0.74**	78.1 ± 0.98**	49.76 ± 1.58
SaO_2_ (%)	93.94 ± 0.17	86.95 ± 0.26**	81.15 ± 0.26**	71.75 ± 0.51**	84.06 ± 0.65**
Hemoglobin (g/dL)	179.20 ± 1.93	186.58 ± 4.02**	197.35 ± 2.47**	200.40 ± 1.88**	190.19 ± 1.47
Ejection fraction (%)	76.24 ± 1.06	56.03 ± 1.63*	53.00 ± 1.68*	47.67 ± 17.40*	59.29 ± 16.30
New York Heart Association Heart Function Rating					
I	50	1			50
II		1	4	7	12
III		18	31	28	78
IV		20	5	5	30

* *P* < .05

** *P* < .01, compared between groups.

### 2.3 Diagnostic criteria for chronic HAPH

Adopted the Qinghai Standard for Chronic Mountain Disease, published by the International Society of Alpine Medicine (ISMM) in June 2005, based on the European Heart Association and the European Respiratory Diseases Association and approved by the International Cardiopulmonary Transplant Association. The Guidelines for the Diagnosis and Treatment of High Pressures define the standard for pulmonary hypertension as the mean pulmonary artery pressure > 25 mm Hg^3^, ^4^ and long‐term residence in high altitude areas. There are clinical manifestations of hypoxemia, such as decreased SaO_2_. There were hypoxic pulmonary hypertension in different degrees. Clinical examination showed Loud pulmonary valve second heart sound. Echocardiography showed pulmonary hypertension. Other diseases leading to pulmonary hypertension were excluded.

### 2.4 Echocardiographic exam

A Phillips EPIQ 7C (coPHILIPS) echocardiographic diagnostic system with probe frequencies ranging from 1 to 5 MHz was used to measure tricuspid regurgitation flow to calculate pulmonary systolic pressure (PASP). The grouping of the included subjects was determined by the measured values, and HAPH was classified into mild (30 to 49 mm Hg [1 mm Hg = 0.133 kPa]), moderate (50 to 69 mm Hg) and severe (≥70 mm Hg).

Left ventricular ejection fraction (LVEF) was derived as follows: LVEF = (EDV‐ESV/EDV) × 100, where EDV and ESV are end diastolic volume and end systolic volume, respectively.

### 2.5 HRV exam

Subject information was collected using a 12‐lead 24‐hour full information DCG system (Holter, Del Mar Reynolds Medical, Lifecard CF). Sampling requirements were: starting point at 9‐11 am and rest for 15 minutes before inspection. The DCG was recorded and replayed on a computer, excluding ventricular/supraventricular premature or other arrhythmias. The 24‐hour continuous DCG was recorded, and the ventricular/supraventricular premature contraction and interference were automatically eliminated by the software.

#### 2.5.1 HRV parameters

Time‐domain indicators for 24‐hour long‐range analysis included SD of normal RR intervals (SDNN), SD of 5 minutes average normal RR intervals (SDANN), root mean square of successive difference in RR interval (RMSSD), percentage of RR intervals differing more than 50 ms from the preceding one (PNN50) and HRVTI, also called triangle index.

The frequency domain index was evaluated in a short time interval of 5 minutes. The subjects were arranged in a quiet time position from 9:00 am to 11:00 am, and frequency domain analysis was performed. The fast Fourier transform method (FFT) was employed. Frequency domain included total power (TP), very low frequency (VLF, 0.03‐0.04 Hz), low frequency (LF, 0.04‐0.15 Hz), and high frequency (HF, 0.15‐0.40 Hz).

### 2.6 Statistical methods

Analyses were performed using SPSS (version 25.0). Parameters with normal distribution were expressed as mean ± SD (SD), and count data as percentage or number of cases. Independent samples *t* test was performed to compare group pairs, and ANOVA was carried out to compare multiple groups. The LSD‐ or SNK‐q test was performed for data with homogeneous variance, and nonparametric tests for those with heterogeneous variance. Generalized linear model (GLM) analysis was used to adjust the confounders. The Kruskal‐Wallis test was performed for analysis. Multiple comparisons were performed by the Mann‐Whitney *U* test. For all tests, *P* values < .05 were considered statistically significant.

## 3 RESULTS

### 3.1 Clinical and hemodynamic characteristics of the patients

The average altitude of all subjects was 3821.19 ± 36.81 m, and there was no significant difference between the groups. Blood SaO_2_ at the fingertip was decreased significantly in the HAPH group compared with control patients (*P* < .01). Hemoglobin were increased significantly in the HAPH group compared with control cases (*P* < .01). The HAPH course was 5.00 ± 0.65 years. It is worth noting that the average heart rate decreased progressively in with increasing HAPH severity (*P* < .05). At the same time, left ventricular ejection fraction decreased progressively with increasing pulmonary artery pressure (*P* < .05) (Table 1).

### 3.2 HRV results

After adjustment for oxygen saturation (SaO_2_), time domain indexes such as SDNN, SDANN, RMSSD, PNN50, and HRVTI progressively decreased from mild to severe groups of HAPH patients compared with control values, with statistically significant differences (*P* ≤ .001). Similarly, after adjustment for SaO_2_, frequency domain indexes such as TP, VLF, LF, and HF progressively decreased from mild to severe groups of HAPH patients compared with control values, and the differences were statistically significant (*P* ≤ .001). In patients with mild pulmonary hypertension, the LF/HF ratio was slightly elevated compared with controls (*P* = .03). Meanwhile, this ratio was lowest in patients with moderate pulmonary arterial pressure followed by the severe diseased patients; both groups showed significantly lower LF/HF ratios compared with control values (*P* < .001). These data are detailed in Table 2.

**Table 2:** Associations of HRV indexes with high altitude pulmonary hypertension (HAPH)

Parameters	B	95%CI	*P* value
**Heart rate variability indexes of time domain of Holter**			
PNN50			
SaO_2_	0.569	(0.247, 0.892)	.001
HAPH			
Severe	−28.863	(−36.233, −21.493)	<.001
Moderate	−21.584	(−26.074, −17.095)	<.001
Mild	−12.561	(−15.43, −9.691)	<.001
Control	ref		
SDNN			
SaO_2_	0.947	(0.539, 1.355)	<.001
HAPH			
Severe	−38.214	(−47.546, −28.882)	<.001
Moderate	−28.080	(−33.765, −22.396)	<.001
Mild	−13.884	(−17.517, −10.25)	<.001
Control	ref		
SDANN			
SaO_2_	0.882	(0.509, 1.254)	<.001
HAPH			
Severe	−28.496	(−37.007, −19.986)	<.001
Moderate	−18.536	(−23.72, −13.352)	<.001
Mild	−11.547	(−14.861, −8.234)	<.001
Control	ref		
RMSSD			
SaO_2_	0.884	(0.477, 1.291)	<.001
HAPH			
Severe	−23.976	(−33.278, −14.673)	<.001
Moderate	−20.709	(−26.375, −15.043)	<.001
Mild	−12.606	(−16.227, −8.984)	<.001
Control	ref		
HRVTI			
SaO_2_	0.902	(0.387, 1.417)	.001
HAPH			
Severe	−40.649	(−52.414, −28.883)	<.001
Moderate	−31.003	(−38.169, −23.836)	<.001
Mild	−18.335	(−22.915, −13.754)	<.001
Control	ref		
**Heart rate variability indexes of frequency domain of Holter**			
TP			
SaO_2_	10.991	(5.587, 16.395)	<.001
HAPH			
Severe	−514.518	(−638.074, −390.962)	<.001
Moderate	−352.784	(−428.046, −277.523)	<.001
Mild	−194.383	(−242.489, −146.276)	<.001
Control	ref		
VLF			
SaO_2_	0.697	(0.28, 1.114)	.001
HAPH			
Severe	−40.479	(−50.013, −30.945)	<.001
Moderate	−30.693	(−36.5, −24.885)	<.001
Mild	−15.864	(−19.576, −12.152)	<.001
Control	ref		
LF			
SaO_2_	0.811	(0.414, 1.208)	<.001
HAPH			
Severe	−36.343	(−45.422, −27.265)	<.001
Moderate	−26.074	(−31.604, −20.544)	<.001
Mild	−13.047	(−16.582, −9.513)	<.001
Control	ref		
HF			
SaO_2_	0.593	(0.308, 0.878)	<.001
HAPH			
Severe	−24.859	(−31.374, −18.345)	<.001
Moderate	−17.098	(−21.066, −13.129)	<.001
Mild	−9.935	(−12.472, −7.398)	<.001
Control	ref		
LF/HF ratio			
SaO_2_	<0.001	(0, 0)	.955
HAPH			
Severe	−0.009	(−0.013, −0.005)	<.001
Moderate	−0.010	(−0.012, −0.007)	<.001
Mild	0.002	(0.001, 0.004)	.003
Control	ref		

#### 3.2.1 Correlation between HRV index and elevated pulmonary arterial pressure

The mean values of each group were consistent with normal distribution, and the homogeneity test of variance showed that the variance was not uniform. The box charts of the average values for each index are shown in Figures S1‐10. The Kruskal‐Wallis test was performed, and the results are shown in Table 3, indicating that the difference in HRV between the groups was statistically significant. Next, Kendall Rank correlation coefficients were determined. The results showed that time domain indexes including SDNN, SDANN, RMSSD, PNN50, and HRVTI, and frequency domain factors such as TP, VLF, LF, HF, and LF/HF were negatively correlated with pulmonary arterial pressure changes (*P* < .01) (Table 4).

**Table 3:** Kruskal‐Wallis test

HRV		Kruskal‐Wallis H	df	*P*‐value
Time domain	PNN50	158.07	3	.000
	SDANN	158.04	3	.000
	SDNN	158.11	3	.000
	RMSSD	158.11	3	.000
	HRVTI	158.10	3	.000
Frequency domain	TP	158.10	3	.000
	VLF	158.10	3	.000
	LF	158.10	3	.000
	HF	157.94	3	.000
	LF/HF	134.77	3	.000

**Table 4:** Correlation analysis of HAPH and HRV

Item	*r*	*P*
Time domain		
PNN50	−.867	.000
SDNN (ms)	−.868	.000
SDANN	−.872	.000
RMSSD	−.868	.000
HRVTI	−.870	.000
Frequency domain		
TP (ms^2^)	−.867	.000
VLF (ms^2^)	−.867	.000
LF (ms^2^)	−.867	.000
HF (ms^2^)	−.866	.000
LF/HF	−.411	.000

## 4 DISCUSSION

Individuals living at high altitudes for a long time could have a series of physiological or pathological changes due to low pressure, low oxygen concentration, dry and cold weather, elevated solar radiation and ultraviolet radiation, which affect pulmonary artery pressure. At present, the important role of autonomic nervous regulation in its development is not clear.

HRV is currently the most widely used method for the evaluation of ANF. As a noninvasive quantitative indicator for judging cardiac autonomic nervous activity, HRV has been confirmed by many clinical trials.^5^, ^6^ It is used to evaluate a variety of cardiovascular diseases because of its noninvasive, simple, and reproducible characteristics.^7^ More importantly, HRV is closely related to clinically meaningful outcome variables, such as heart events, morbidity, and mortality.

In the time domain of HRV, HRVTI represents the overall change of heart rate. SDNN and SDANN represent enhanced sympathetic activity. RMSSD and PNN50 represent weakened vagal activity, while RMSSD is more often used to evaluate vagal nerve function because of its good stability. The time domain analysis in this study was performed on a 24‐hour time course, so assessment of long‐term ANF in patients with HAPH is more accurate.

In this study, with the increase of pulmonary artery pressure, all time domain indexes of patients with HAPH were significantly reduced. We observed a significant decrease in ANF in patients with chronic HAPH. HRV frequency domain analysis often uses short‐term studies, so it is more suitable for short‐term evaluation of ANF in patients with HAPH. The TP in the frequency domain represents the sum of high frequency, very low frequency, and ultralow frequency. LF was once used as a very important indicator of HRV to reflect the functional status of the sympathetic nerve. However, with further research, LF is no more considered an effective marker of autonomic nervous activity itself, because besides autonomic nervous system, it is also affected by baroreceptor activity.^8^ HF reflects the excitability of the vagus nerve. LF/HF representing the balance of autonomic nerves has been widely recognized. Short‐term frequency domain analysis in this study showed that the increase of pulmonary artery pressure in patients with HAPH was negatively correlated with the changes of vagus nerve and sympathetic nerve. As pulmonary arterial pressure increased, the function of autonomic nerves was significantly attenuated. LF/HF increased in patients with mild HAPH, suggesting that ANF may be increased in patients with HAPH in the early stage of onset. In patients with moderate HAPH, LF/HF was the lowest, suggesting that autonomic function was significantly inhibited at this stage. There was a small increase in LF/HF in patients with severe HAPH, suggesting that autonomic function may be out of control at this stage.

In addition, studies in China have shown that the incidence of sinus tachycardia in patients with type I pulmonary hypertension mainly including idiopathic pulmonary hypertension, congenital heart disease‐related pulmonary hypertension and connective tissue disease‐related pulmonary hypertension can reach 26%.^9^ According to the 2009 American College of Cardiology Foundation/American Heart Association's consensus on pulmonary hypertension, HAPH belongs to type III pulmonary hypertension.^10^ In this study, we found that the average heart rate of patients with HAPH was significantly reduced, and this decrease was more pronounced with increasing pulmonary artery pressure. This may be related to the fact that the subjects selected were Tibetans living at high altitudes. Their adaptation to high altitude has obvious genetic basis. In the process of adapting to hypoxic and hypobaric natural environment, their dominant physiological adaptation is no longer the enhancement of respiratory and circulatory functions, but more dependent on organizational adaptation. The Tibetans’ ability to carry oxygen in circulation is enhanced, and oxygen is used more economically and effectively. Therefore, the compensatory increase of heart rate is not needed. Previous studies of healthy Tibetan adults living in Qinghai have also confirmed this point.^11^

### 4.1 Limitations

We used noninvasive methods to assess pulmonary artery pressure and cardiac function in this study. These methods tend to underestimate the level of pulmonary arterial pressure, which is not as accurate as invasive measurement methods.^12^ For ethical reasons, invasive measurement methods cannot be used in healthy individuals. Although the effects of drugs and other factors have been excluded, in addition to the effects of HAPH, the effect of hypoxia on HRV cannot be ruled out, and the complexity of the relationship between the two is often inconsistent. Due to the complexity of the relationship between hypoxia and HRV, inconsistent results are often found in the literature.^13^, ^14^, ^15^, ^16^ However, patients with chronic HAPH with an average duration of 5.00 (+0.65) years were selected in this study because they had achieved long‐term adaptation to hypoxia. In general, hypoxia is considered to be effective in stimulating the sympathetic nervous system, resulting in a decrease in HRV variability (LF and HF components) and an increase in LF/HF ratios, which is inconsistent with the results of this study and fully demonstrates hypoxia in chronic altitude sickness. In this study, due to the limited sample size, we could not assess associations of HRV with hemoglobin levels and SaO_2_. Further validation studies are needed to verify these findings.

## CONFLICT OF INTEREST

The authors have declared that no potential conflicts of interest exist with any companies/organizations whose products or services may be discussed in this article.

## Supporting information


**Figures S1‐10** The **box** charts of the average values of various HRV indexes.
