# Supplementary figures and images for: Retrospective cohort analysis of heart rate variability in patients with high altitude pulmonary hypertension in Tibet
Source: Clin Cardiol. 2019 Dec 19;43(3):298–304. doi: 10.1002/clc.23312 (PMC7068065; doi:10.1002/clc.23312)

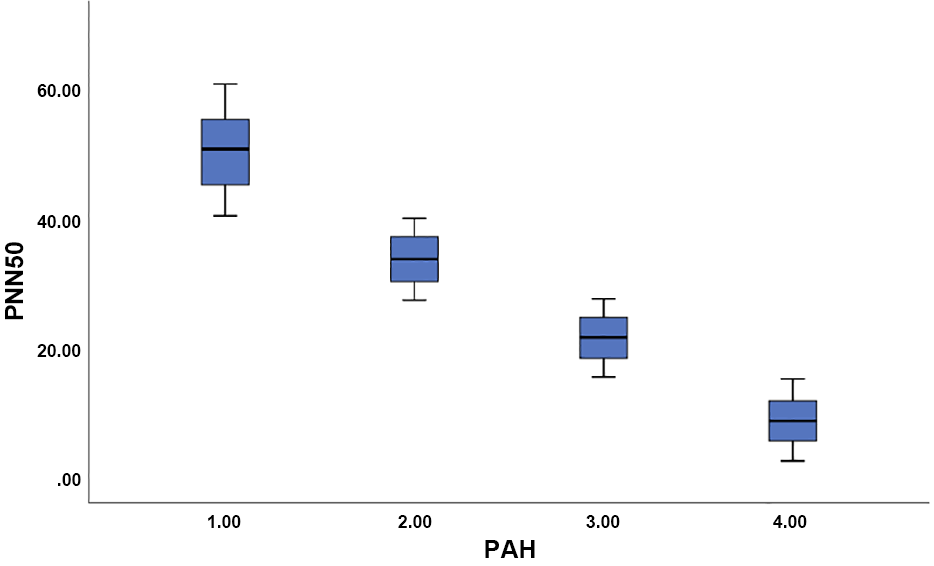

Supplement: Supplementary file 1 — Figures S1‐10 The box charts of the average values of various HRV indexes. [file CLC-43-298-s001.zip › clc23312-sup-0001-FigureS1.tif]

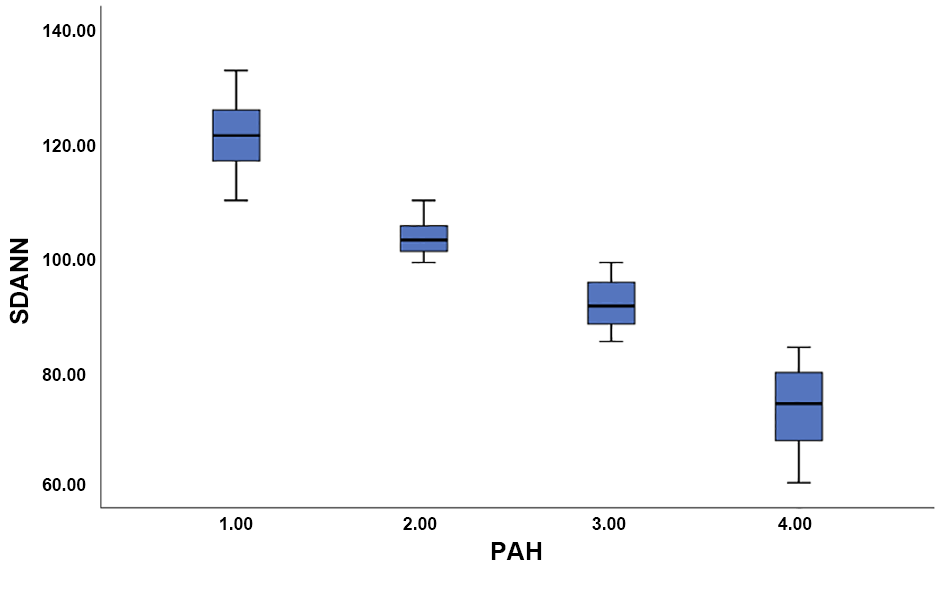

Supplement: Supplementary file 1 — Figures S1‐10 The box charts of the average values of various HRV indexes. [file CLC-43-298-s001.zip › clc23312-sup-0002-FigureS2.tif]

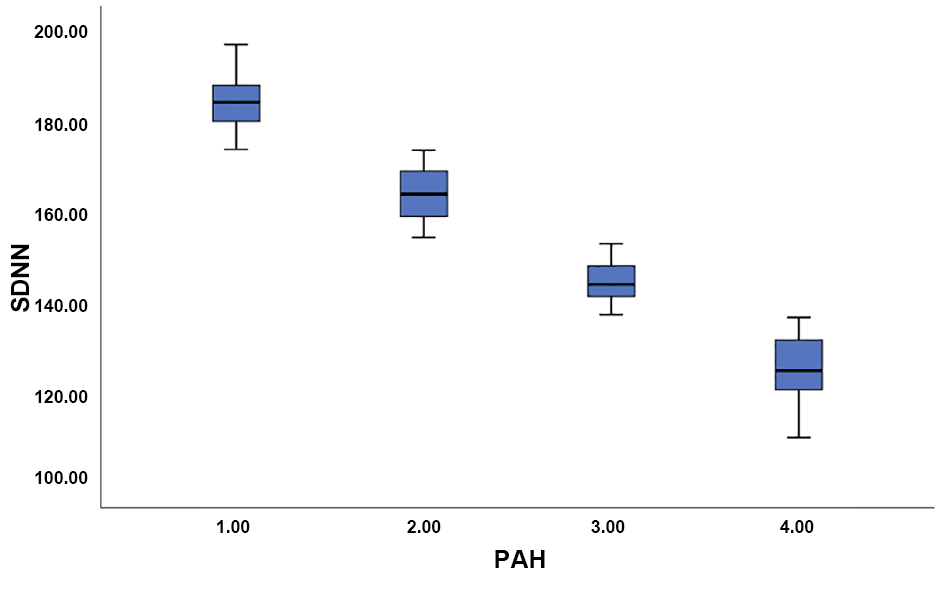

Supplement: Supplementary file 1 — Figures S1‐10 The box charts of the average values of various HRV indexes. [file CLC-43-298-s001.zip › clc23312-sup-0003-FigureS3.tif]

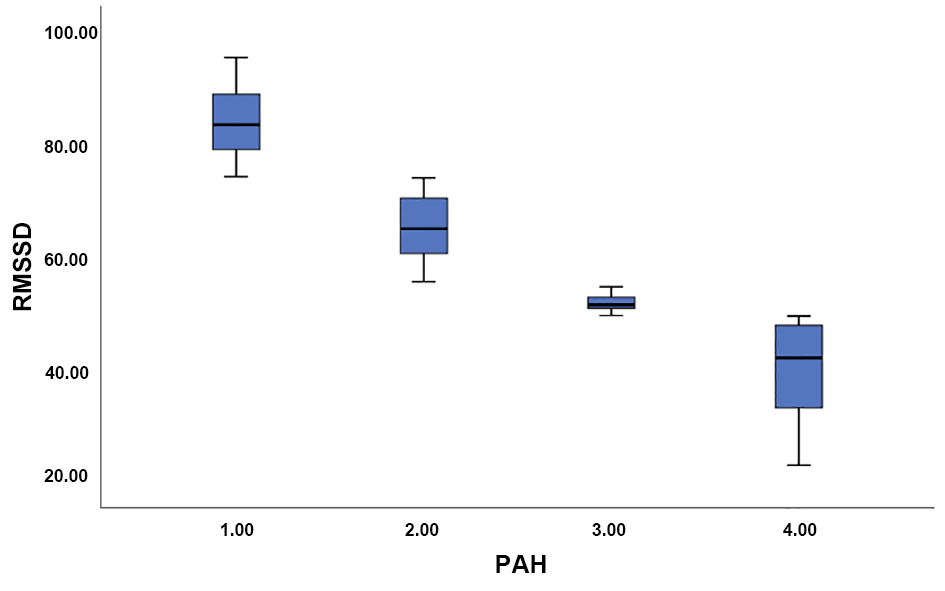

Supplement: Supplementary file 1 — Figures S1‐10 The box charts of the average values of various HRV indexes. [file CLC-43-298-s001.zip › clc23312-sup-0004-FigureS4.tif]

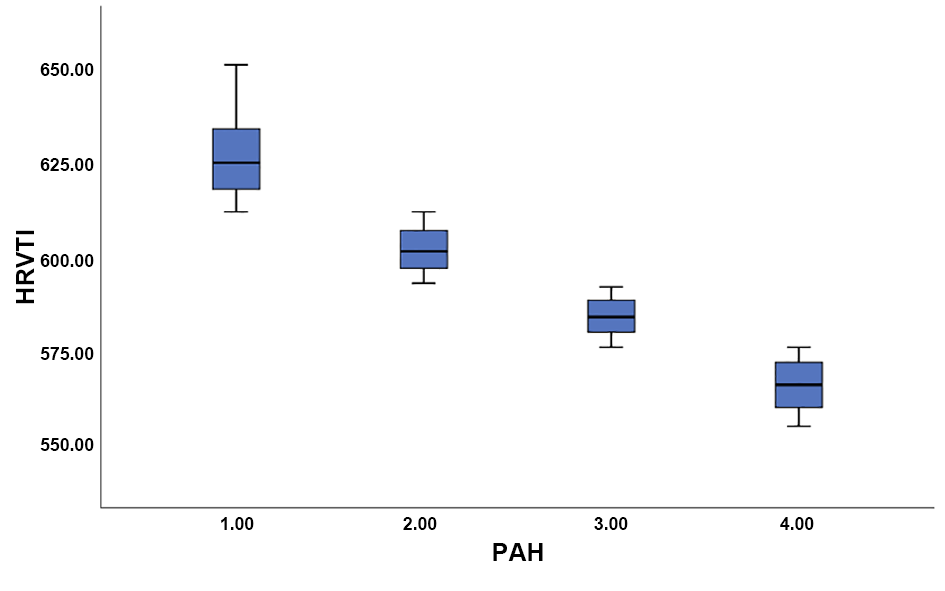

Supplement: Supplementary file 1 — Figures S1‐10 The box charts of the average values of various HRV indexes. [file CLC-43-298-s001.zip › clc23312-sup-0005-FigureS5.tif]

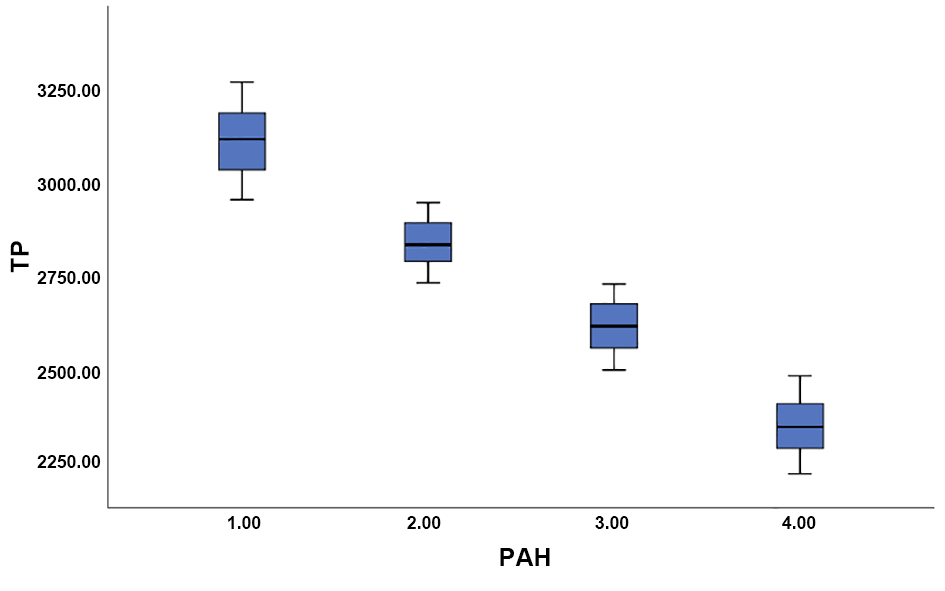

Supplement: Supplementary file 1 — Figures S1‐10 The box charts of the average values of various HRV indexes. [file CLC-43-298-s001.zip › clc23312-sup-0006-FigureS6.tif]

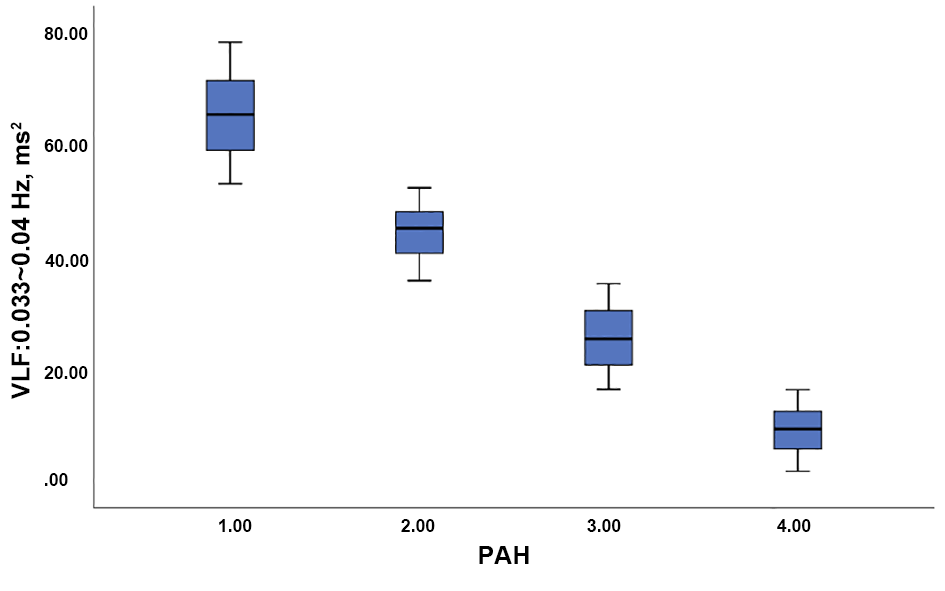

Supplement: Supplementary file 1 — Figures S1‐10 The box charts of the average values of various HRV indexes. [file CLC-43-298-s001.zip › clc23312-sup-0007-FigureS7.tif]

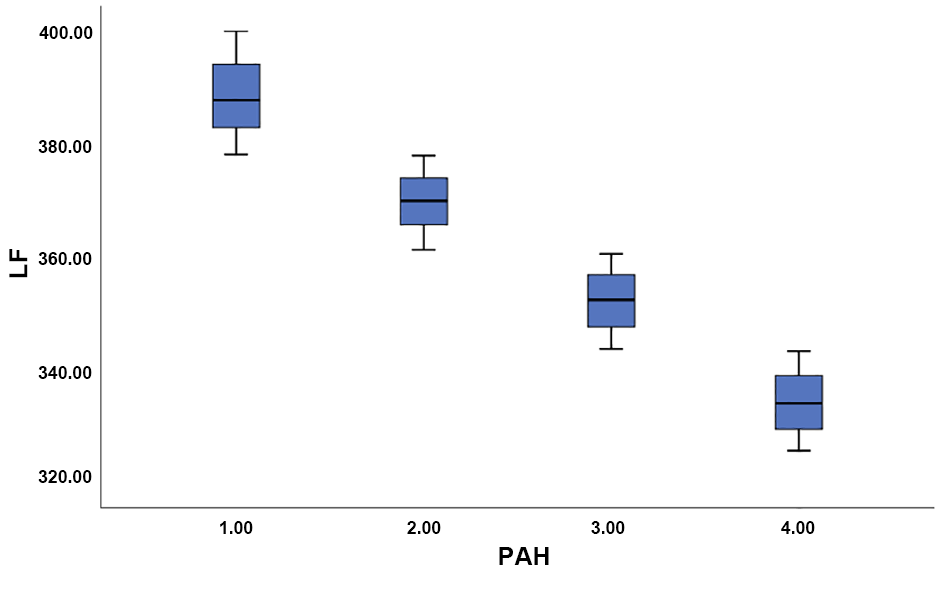

Supplement: Supplementary file 1 — Figures S1‐10 The box charts of the average values of various HRV indexes. [file CLC-43-298-s001.zip › clc23312-sup-0008-FigureS8.tif]

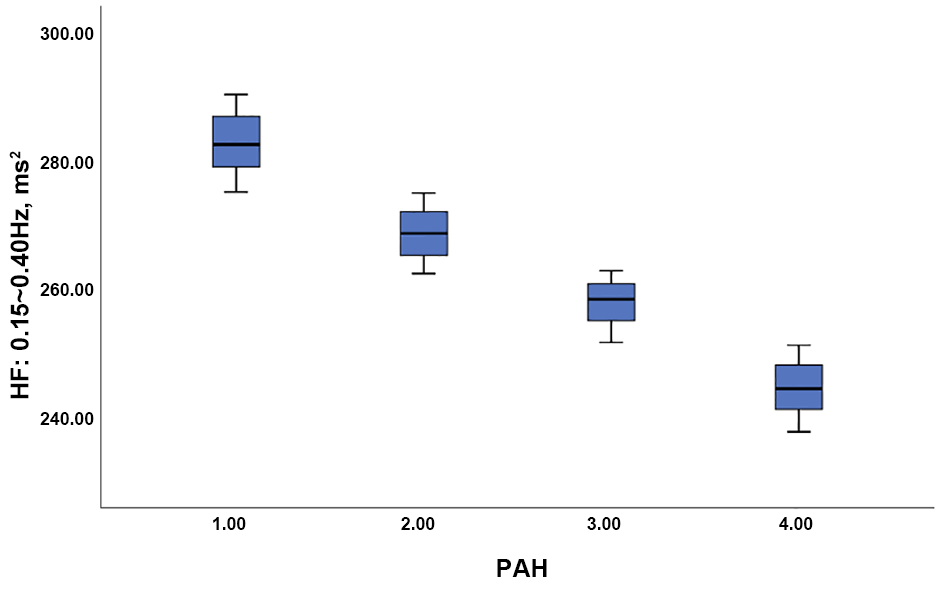

Supplement: Supplementary file 1 — Figures S1‐10 The box charts of the average values of various HRV indexes. [file CLC-43-298-s001.zip › clc23312-sup-0009-FigureS9.tif]

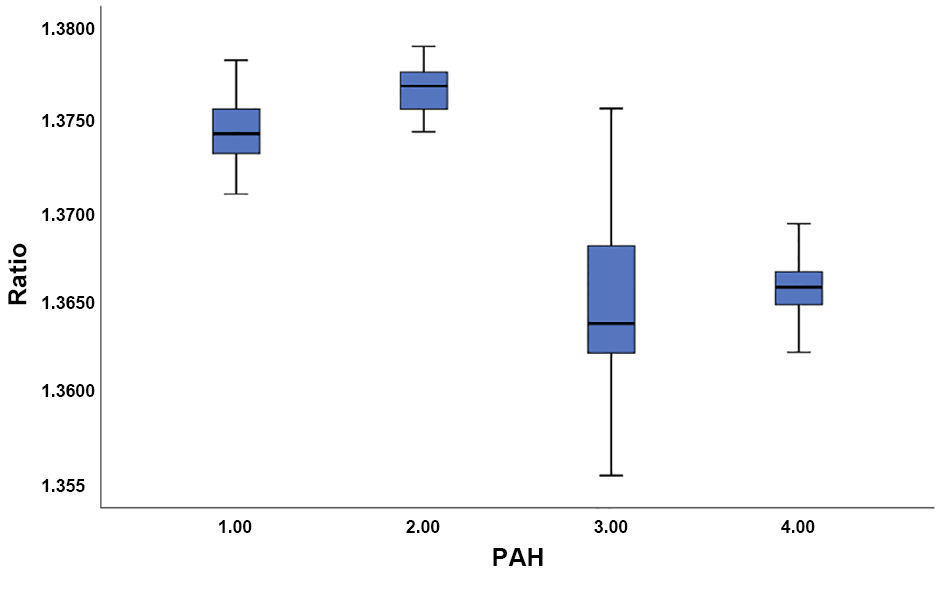

Supplement: Supplementary file 1 — Figures S1‐10 The box charts of the average values of various HRV indexes. [file CLC-43-298-s001.zip › clc23312-sup-0010-FigureS10.tif]
